# Ricord’s Letters on Syphilis

**Published:** 1853-01

**Authors:** 


RICORD'S LETTERS ON SYPHILIS.

Our readers need not to be told that Ricord is the great authority on *Syphilis*. He has devoted himself almost exclusively to its investigation and treatment during his whole professional life under circumstances the most favorable to successful inquiry. He has seen the disease in all its varied aspects, daily and hourly, in hospital and private practice, for now many years. He has moreover all this time been a scientific investigator of the affection. He has looked at it with a curious eye and a resolute purpose to understand its nature, history, mode of propagation, and all the phenomena pertaining to it. That he has, therefore, attained a knowledge of the disorder exceeding that of his predecessors, and, perhaps, all his contemporaries, is what is to be expected. At an early day we purpose to give our readers some account of these "Letters," containing the author's latest views on the subject.

